# Motion Analysis and Tactile-Based Impedance Control of the Chest Holder of a Piggyback Patient Transfer Robot

**DOI:** 10.1155/2021/9918019

**Published:** 2021-07-19

**Authors:** Yuxin Liu, Yuting Yin, Zhiwen Jiang, Shijie Guo

**Affiliations:** ^1^State Key Laboratory of Reliability and Intelligence of Electrical Equipment, Hebei University of Technology, Tianjin 300130, China; ^2^Hebei Key Laboratory of Robot Sensing and Human-Robot Interaction, Tianjin 300130, China; ^3^School of Mechanical Engineering, Hebei University of Technology, Tianjin 300130, China

## Abstract

Patient transfer, such as carrying a bedridden patient from a bed to a pedestal pan or a wheelchair and back, is one of the most physically challenging tasks in nursing care facilities. To reduce the intensity of physical labor on nurses or caregivers, a piggyback transfer robot has been developed by imitating the motion when a person holds another person on his/her back. As the chest holder supports most of the weight of the care-receiver during transfer, a human-robot dynamic model was built to analyze the influences of the motion of the chest holder on comfort. Simulations and experiments were conducted, and the results demonstrated that the rotational motion of the chest holder is the key factor affecting comfort. A tactile-based impedance control law was developed to adjust the rotational motion. Subjective evaluations of ten healthy subjects showed that adjusting the rotational motion of the chest holder is a useful way to achieve a comfortable transfer.

## 1. Introduction

With the advent of an ageing society [1–5], the demand for nursing care robots that can help on-site caregivers is increasing. Among nursing care tasks, patient transfer, such as carrying a bedridden patient from a bed to a wheelchair and back, is one of the most physically challenging tasks in nursing care. It is a direct cause of disabling musculoskeletal disorders (MSDs) in caregivers [6]. Therefore, many kinds of transfer devices [7, 8] have been proposed and developed to provide comfort and safe transfer. These devices include Yaskawa transfer device [9], Panasonic integrated bedchairs [10], and the Robohelper Sasuke [11]. Although some transfer devices have been commercialized, they are not widely used in nursing care facilities. These devices require considerable time and space to perform a transfer task. In addition, there are still problems with safety, convenience, and comfort. It was reported that a caregiver's physical burden could not be reduced in many cases by using transfer lifts [12]. To address saving time, dual-arm care robots, such as the RoNA (Robotic Nursing Assistant System) [13] and RIBA (Robot for Interactive Body Assistance) [14], have been developed. These robots have human-type arms for carrying and moving a care-receiver from a bed to a wheelchair and back. However, they are expensive and complicated to operate, making them inconvenient for practical use.

**Figure 1:**
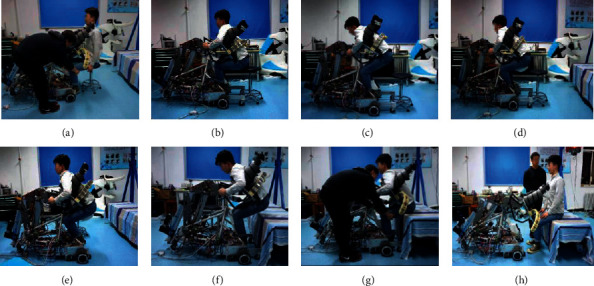
Transfer motion of the proposed robot.

To solve the above problems, the authors' group developed a transfer robot that had a more flexible and simpler configuration. The robot was designed by imitating the motion when a person holds another person on his/her back, as shown in Figure 1. Some research institutions, such as Toyota Motors [15] and Fuji Machinery Co., Ltd. [16], have been developing transfer robots based on the same concept. However, these robots have only two degrees of freedom in the mechanism of chest support, making them uncomfortable. To make the robot safer and more comfortable, the chest holder of our robot had three degrees of freedom, and it could carry a care-receiver similar to the motion of a person holding up another person on his/her back. Furthermore, the robot had hip support that could automatically spread out to support the care-receiver when the robot moved after holding up a care-receiver [17].

Care-receiver comfort is an essential consideration for a transfer device. Most devices only use passive cushions [18, 19], such as sponge mats, to increase comfort and prevent harm to the care-receiver. Although this can help to reduce the impact and pressure concentration acting on the body of a care-receiver, the passive cushion deformation range is limited. It is difficult to make a robot adapt to the body of the care-receiver and to change posture softly by using only a passive cushion. Since these robot motions have been designed in advance, they cannot be adjusted when the care-receiver has a posture that changes suddenly during carrying. Thus, the motions of the robot should be adjusted during carrying.

To achieve a comfortable carrying motion for the patient transfer robot developed by the authors' group, a tactile-based motion adjustment approach was proposed and introduced to the robot. A dynamic model of the human-robot system was also built. Experimental results for 10 subjects demonstrated that the robot using the adjustment motion approach can carry a care-receiver more comfortably than before.

The paper is organized as follows.  Section 2 describes the configuration of the patient transfer robot, including the mechanical structure and specifications.  Section 3 introduces the dynamic model and demonstrates that the angle adjustment of the chest holder is the most effective way to achieve a comfortable transfer for a care-receiver by using dynamic simulation and six subject experiments.  Section 4 presents the motion adjustment approach, the experimental design, and the test results.  Section 5 covers the conclusions and a brief explanation of future work.

## 2. The Piggyback Transfer Robot

**Figure 2:**
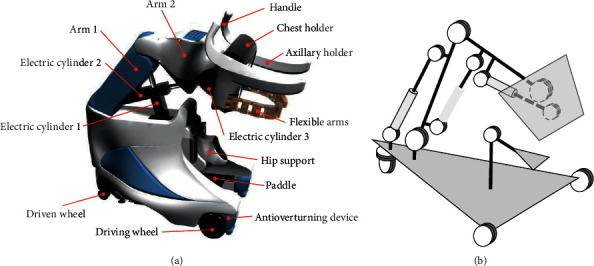
Structure of the piggyback transfer robot. (a) The piggyback transfer robot. (b) Joint configuration of the robot.

The configuration of the piggyback transfer robot is shown in Figure 2, and its basic specifications are summarized in Table 1. The chest holder is the most important part of the robot and can carry a person similar to how a person holds another person by using a hybrid structure. It is driven by three electric cylinders and has three degrees of freedom, including rotation and movement in the horizontal and vertical directions. Although the motion of the whole robot on its wheel can work to compensate for horizontal motions of the chest holder kinematically, such a freedom degree is still required in the chest holder since the robot's heavyweight makes it difficult to drive the robot at a high response. During transfer, a care-receiver is carried up by supporting his/her chest and armpits with the chest holder and axillary holders, respectively. In addition, flexible arms hold the care-receiver tightly to prevent him/her from falling off the robot. As shown in Figure 1, most of the weight of the person who is lifted is supported by the chest holder in carrying. To ensure the safety and comfort of the lifted person, the chest holder, axillary holders, and flexible arms are covered with polyurethane foam (see Figure 2(a)).

The basic motions of the robot are created by interpolating several postures designated in advance. The motions that involve different persons, however, need adjustment to the actual situation. The previous test results of the prototype showed that the robot could carry and move a person from a chair to a toilet pan or a bed and back.

## 3. Motion Analysis of the Chest Holder

### 3.1. Human-Robot Dynamic Model

**Figure 3:**
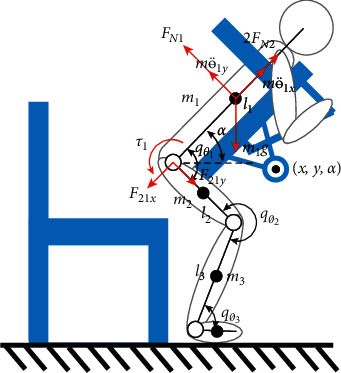
Human-robot dynamic model.

The force between a care-receiver and the robot is the main factor affecting care-receiver's comfort. To analyze the relationship between the forces exerted on the care-receivers and the posture of the robot during carrying, a dynamic model of the care-receiver and robot is established and shown in Figure 3. In this model, a care-receiver is regarded as a four-link, which has a uniformly distributed mass, to simplify the complex situation in actual carrying. In carrying, the care-receiver's chest is in full contact with the chest holder and moves with it. The forces acting on a care-receiver vary with the posture of the chest holder, including the change in horizontal and vertical displacement and angle variation. The position and posture of the chest holder are represented by (*x*, *y*, *α*), where *x* is the horizontal distance between the joint of the chest holder and the origin of the world frame, *y* is the vertical distance between the chest holder's joint and the origin of the world frame, and *α* is the angle between the chest holder and the ground.

During carrying, a care-receiver puts his/her foot on the robot pedal. His/her feet do not move relative to the robot pedal. The center of the ankle joint is regarded as the origin of the global coordinate system. The positions of the center of mass of the care-receiver's torso, thigh, and calf are

(1) $$\begin{array}{c} \left\{\begin{array}{c} x_{3}=\frac{1}{2}\;l_{3}\cos\;q_{\theta_{3}}\left(x,y,\alpha \right),\\ y_{3}=\frac{1}{2}\;l_{3}\sin\;q_{\theta_{3}}\left(x,y,\alpha \right), \end{array}\right.\\ \left\{\begin{array}{c} x_{2}=l_{3}\cos\;q_{\theta_{3}}\left(x,y,\alpha \right)+\frac{1}{2}\;l_{2}\cos\left(q_{\theta_{3}}\left(x,y,\alpha \right)+q_{\theta_{2}}\left(x,y,\alpha \right)\right),\\ y_{2}=l_{3}\sin\;q_{\theta_{3}}\left(x,y,\alpha \right)+\frac{1}{2}\;l_{2}\sin\left(q_{\theta_{3}}\left(x,y,\alpha \right)+q_{\theta_{2}}\left(x,y,\alpha \right)\right), \end{array}\right.\\ \left\{\begin{array}{c} x_{1}=l_{3}\cos\;q_{\theta_{3}}\left(x,y,\alpha \right)+l_{2}\cos\left(q_{\theta_{3}}\left(x,y,\alpha \right)+q_{\theta_{2}}\left(x,y,\alpha \right)\right)+\frac{1}{2}\;l_{1}\cos\left(q_{\theta_{3}}\left(x,y,\alpha \right)+q_{\theta_{2}}\left(x,y,\alpha \right)+q_{\theta_{1}}\left(x,y,\alpha \right)\right),\\ y_{1}=l_{3}\sin\;q_{\theta_{3}}\left(x,y,\alpha \right)+l_{2}\sin\left(q_{\theta_{3}}\left(x,y,\alpha \right)+q_{\theta_{2}}\left(x,y,\alpha \right)\right)+\frac{1}{2}\;l_{1}\sin\left(q_{\theta_{3}}\left(x,y,\alpha \right)+q_{\theta_{2}}\left(x,y,\alpha \right)+q_{\theta_{1}}\left(x,y,\alpha \right)\right), \end{array}\right. \end{array}$$

where *l*_1_, *l*_2_, and *l*_3_ are the lengths of the torso, thigh, and calf of a care-receiver. The angles of the hip, knee, and ankle joints are *q*_*θ*_1__(*x*, *y*, *α*), *q*_*θ*_2__(*x*, *y*, *α*), and *q*_*θ*_3__(*x*, *y*, *α*). The angle of the torso is *α*=*q*_*θ*_1__(*x*, *y*, *α*)+*q*_*θ*_2__(*x*, *y*, *α*)+*q*_*θ*_3__(*x*, *y*, *α*)+2*π*. The kinetic energy of each part is

(2) $$\begin{array}{c} K_{i}=\frac{1}{2}\;m_{i}{\left(\frac{l_{i}}{2}\right)}^{2}{q_{\theta_{i}}}^{2}\left(x,y,\alpha \right)+\frac{1}{2}\;I_{i}{q_{\theta_{i}}}^{2}\left(x,y,\alpha \right), \end{array}$$

where *I*_*i*_ is the inertia tensor. The potential energy is

(3) $$\begin{array}{c} P_{i}=-m_{i}gy_{i}. \end{array}$$

The care-receiver's Lagrangian dynamic equation is

(4) $$\begin{array}{c} L=K-P=\overset{3}{\underset{i=1}{\sum }}K_{i}+\overset{3}{\underset{i=1}{\sum }}P_{i}, \end{array}$$

where the Lagrange operator is *L*. The care-receiver torso's Lagrangian dynamic equation is

(5) $$\begin{array}{c} \tau_{1}=\frac{\text{d}}{\text{d}t}\frac{\partial L}{\partial q_{\theta_{1}}\left(x,y,\alpha \right)}-\frac{\partial L}{\partial q_{\theta_{1}}\left(x,y,\alpha \right)}, \end{array}$$

where the care-receiver's hip joint torque is *τ*_1_.

The friction between a care-receiver and the robot is not considered in this model. The care-receiver's chest and armpits are supported by the robot's chest holder and armpit supports, respectively. There is no relative movement between the care-receiver and the robot. As shown in Figure 3, the care-receiver's torso gravity is *m*_1_*g*, the force between the torso and legs are *F*_21*x*_ and *F*_21*y*_, respectively, the force acting on the care-receiver's armpits is 2*F*_*N*2_, and the force acting on the chest of a care-receiver is *F*_*N*1_. According to the force and moment balance equation, the forces acting on the care-receiver's chest and an armpit *F*_*N*1_*F*_*N*2_  are obtained.

(6) $$\begin{array}{c} \left\{\begin{array}{c} F_{N1}-m_{1}g\;\cos\;\alpha -F_{21y}=m_{1}q_{\theta_{1}y}\left(\ddot{x},y,\alpha \right),\\ 2F_{N2}-m_{1}g\;\sin\;\alpha -F_{21x}=m_{1}q_{\theta_{1}x}\left(\ddot{x},y,\alpha \right),\\ \frac{l_{1}}{2}\;F_{N1}-\frac{l_{1}}{2}\;m_{1}g\;\sin\;\alpha =\tau_{1}, \end{array}\right. \end{array}$$

where the force between the torso and legs *F*_21*x*_, *F*_21*y*_ can be obtained by balancing the forces and torques of each part of the lower limbs.

### 3.2. Dynamic Simulation and Experiment

A dynamic simulation is performed in ADAMS to analyze the effect of the chest holder's motion on the force exerted on a care-receiver. The chest holder's motion, including horizontal and vertical displacement and angle variation, directly affects the forces exerted on a care-receiver.

**Figure 4:**
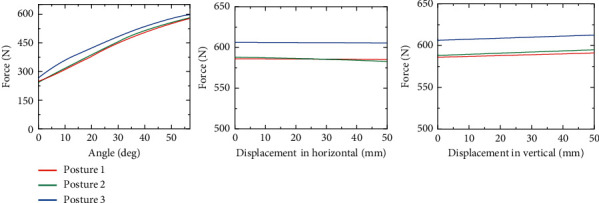
The force acting on the model's chest in simulation.

In the simulation, the chest holder's horizontal and vertical displacement and angle are adjusted individually to find the most significant factors affecting the force exerted on a care-receiver. Three chest holder postures are selected as the initial postures for individual adjustment. These postures, shown in Figure 4(b), divide the carrying motion into four stages. The range of displacement adjustment is set to 0–50 mm, and the range of angle adjustment is set to 0–57 deg. In addition, the influence of the care-receiver's height on the force between the human and robot is also considered. Three models with the same weight and different heights are established, as shown in Table 2.

**Figure 5:**
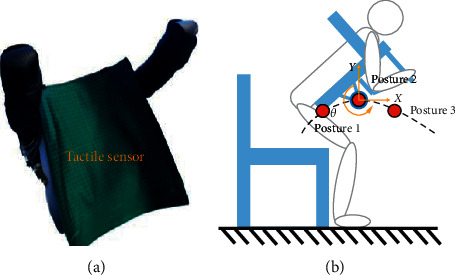
The experiment design of motion adjustment. (a) Chest holder with a tactile sensor. (b) Schematic diagram of robot's motion adjustment in simulation and experiment.

Figure 5 shows the simulation result. This result is an average of three models since there are few obvious differences in the forces on the models of different heights. The result reflects that the force acting on the care-receivers' chest has the most obvious variation with the angle adjustment of the chest holder. Therefore, it is considered that the chest holder's angle adjustment is the main factor affecting the force exerted on the care-receiver. Furthermore, the care-receiver's height does not need to be considered when analyzing the factors affecting the force exerted on the care-receiver.

To confirm the simulation results, an experiment was also conducted. Six subjects, shown in Table 2, participated in this experiment. In the experiment, a tactile sensor (Tekscan CONFOR Mat (Map #5330E) [20]) was mounted on the chest holder to detect the force acting on the care-receiver during carrying. Tekscan pressure sensors are used in biomechanics research to measure contact loads since they are thin (2 mm) and soft and offer high resolution and straightforward data acquisition. The sensor consists of 32 pixels × 32 pixels, and the chest pressure at 1024 individual pressure-sensing locations can be measured simultaneously with minimal error. Its pressure range is 0–580 mmHg and capturing frequency is 100 Hz. During experiment, all subjects were asked to be in a completely relaxed state in carrying, except for grabbing the robot handles with hands, to avoid the influence of the active motion of the subjects on the force. The robot motion in the experiment was completely consistent with the motion in the simulation.

**Figure 6:**
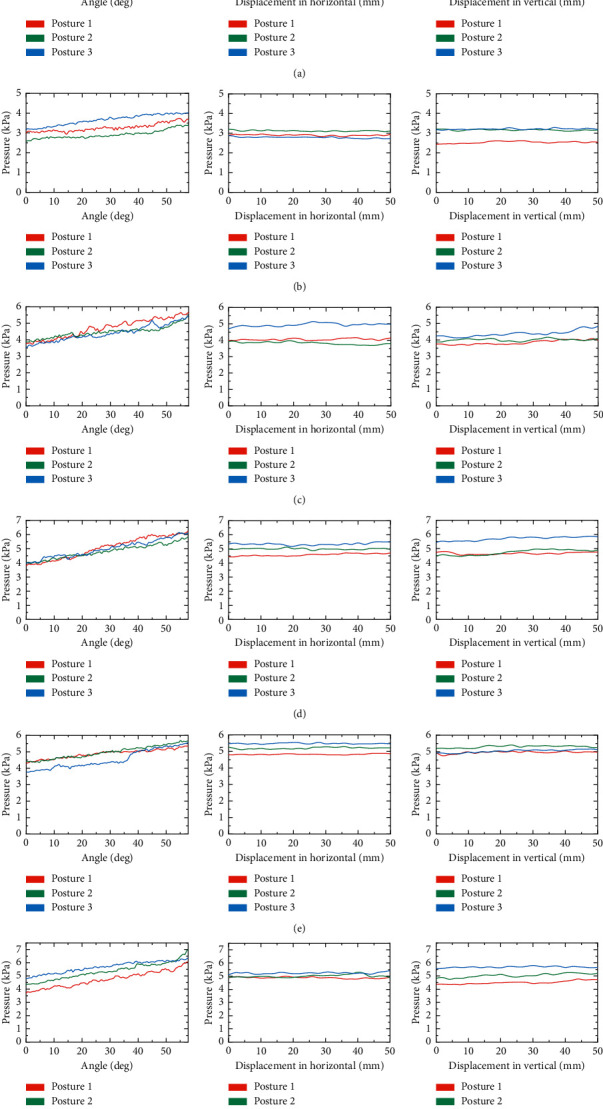
The force (pressure) acting on subjects' chest in the experiment. (a) Subject 1. (b) Subject 2. (c) Subject 3. (d) Subject 4. (e) Subject 5. (f) Subject 6.

As illustrated in Figure 6, the change trends of the force acting on subjects in the experiment are consistent with the simulation result. The force exerted on the care-receiver has the most obvious change with the chest holder's angle adjustment. Therefore, the comfort of care-receivers can be ensured by adjusting the angle of the chest holder. It should be noted that the position of the pressure center of the care-receiver's chest does not change significantly with the individual displacement and angle adjustment of the chest holder in the simulation and experiment. Therefore, the variation in the torque acting on the chest holder's chest with motion adjustment is not indicated in the description of the simulation and experimental results.

## 4. Tactile-Based Impedance Control of the Chest Holder and Experiment

The basic motions of the robot have been created by interpolating several designated postures. Although the motions are designed according to the comfort of the care-receiver, they cannot ensure the care-receiver's comfort when the care-receiver's posture changes suddenly during carrying. Thus, the robot motions may need adjustment.

### 4.1. Tactile-Based Motion Adjustment

**Figure 7:**
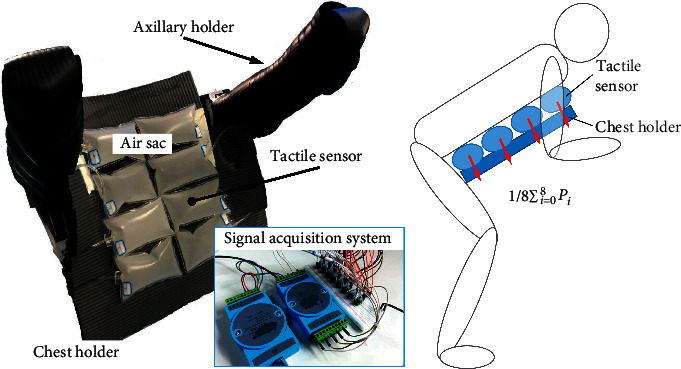
The tactile sensor mounted on the chest holder.

The detection of the force acting on the body of the care-receiver was necessary for the motion adjustment of the robot. The chest holder's posture can be adjusted according to the actual force to ensure the comfort of the care-receiver. A tactile sensor consisting of air sacs and air pressure sensors that was similar to a cushion was installed on the surface of the chest holder to detect the force acting on the chest of the care-receiver and is depicted in Figure 7. The sensor measured the force acting on the body of the care-receiver by using the air pressure sensor to detect the air pressure change in the air sac.

The force acting on the care-receiver is also the most significant factor affecting the comfort of the care-receiver. An active impedance control [21–29] method was proposed to ensure the care-receiver's comfort. The control method could adjust the angle of the chest holder according to the deviation between the actual force acting on the care-receiver's chest *P*_ext_(*t*) and the force, making the care-receiver comfortable *P*_ref_. The extension and retraction of the electric cylinder 3 caused the chest holder to rotate around its joint. The extension of the electric cylinder 3 decreased the angle of the chest holder and conversely increased the angle of the chest holder. Therefore, the angle of the chest holder would be adjusted through the extension and retraction of the electric cylinder 3, when there was a deviation between *P*_ext_(*t*) and *P*_ref_. The model of the control method was

(7) $$\begin{array}{c} P_{\text{ext}}\left(t\right)-P_{\text{ref}}={M\;}_{d}\Delta \ddot{X}\left(t\right)+{B\;}_{d}\Delta \dot{X}\left(t\right)+{K\;}_{d}\Delta X\left(t\right), \end{array}$$

(8) $$\begin{array}{c} X_{\text{ref}}\left(t\right)={\hat{X}}_{\text{ref}}\left(t\right)+\Delta X\left(t\right). \end{array}$$

The calculation of (7) was actually performed at discrete sampling times *t*. In this equation, *M* _*d*_, *B* _*d*_, and *K* _*d*_ are the virtual inertia, viscosity, and stiffness, respectively. *X*_ref_(*t*) is the designed displacement of the electric cylinder 3. ${\hat{X}}_{\text{ref}}\left(t\right)$ is an adjustment for *X*_ref_(*t*) to ensure the care-receiver's comfort. *P*_ext_(*t*) is the actual force acting on the care-receiver's chest. This can be obtained by using the tactile sensor. The values of *M* _*d*_, *B* _*d*_, *K* _*d*_, and *P*_ref_ were important to achieve a comfortable lift for a care-receiver and were designed in advance by testing. The control variate method was used to determine the values of *M* _*d*_, *B* _*d*_, and *K* _*d*_ in the simulation and the actual testing. Appropriate values of *M* _*d*_, *B* _*d*_, and *K* _*d*_ reduce the amount of oscillation and overshoot in the chest holder system and make the system be stabilized more quickly. In addition, the stiffness coefficient *K* _*d*_ has a significant effect on the chest holder system with a low speed and the comfort of the care-receiver. The determination of *K* _*d*_ needs to consider the subjective feelings of the care-receiver, compared with *M* _*d*_ and *B* _*d*_. *P*_ref_ is also determined according to the feelings of the care-receiver in advance, and its value is different for different care-receivers. The previous test results [30] of the robot showed little difference in both forces acting on the care-receiver chest when the care-receivers were being carried in a comfortable posture. It was feasible that *P*_ref_ could be regarded as a constant value. In addition, the values of *P*_ref_ are also different for different care-receivers. This needed to be determined for different care-receivers in advance. The control method could offer a care-receiver a comfortable lift when the actual force was equal to this reference pressure. If the actual force was excessive, the chest holder's angle had to be decreased; conversely, the angle had to be increased.

### 4.2. Experiment

An experiment considering the care-receiver's subjective feelings was conducted to verify that the motion adjustment method is effective in ensuring the comfort of the care-receiver. Ten experimenters (see Table 3) participated in the experiment. The experiment was conducted in the morning to avoid the impact of a full abdomen on the lifting motion. Additionally, the experimenters was asked to have a full rest and fast within two hours before the experiment. To prevent the impact of physical fatigue caused by the carrying motion, the experimenters were also given sufficient rest after each experimental step.

In this experiment, the experimenters were carried out by using the original basic motion and the adjusted motion. Different experimenters have different feelings due to their height, weight, and gender. Therefore, the basic motion of each experimenter was individually designed based on their feelings of each experimenter in advance to ensure their comfort. In carrying with the adjusted motion, the angle adjustment range of the chest holder depends on the impedance parameters. According to the simulation and test, the active impedance control parameters are summarized in Table 4. The prerecorded values of the force making the care-receiver comfortable *P*_ref_ were determined in carrying based on the subjective feelings of the experimenters. The optimal value from many test results was chosen to ensure the comfort of the care-receiver as much as possible. To ensure the comfort of the experimenter, *P*_ref_ for each experimenter was individually designed according to their subjective feelings. In principle, these parameters were set tentatively for the experiments with the experimenters and should be optimized for humans in future experiments. The motion of the chest holder, the force acting on the care-receiver's chest, and the feeling of experimenters were recorded.

**Figure 8:**
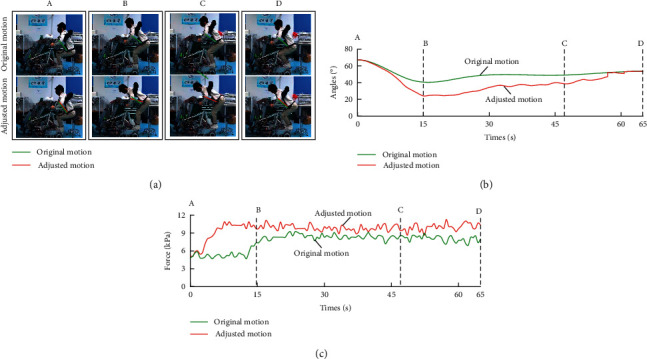
Experimental results of motion adjustment. (a) Comparison of original and adjusted motions. (b) Angle of the chest holder. (c) Force of the experimenter's chest.

A healthy experimenter's test (height 175 cm, weight 60 kg) result is shown as an example in Figure 8. During carrying, the angle of chest holder was obviously adjusted when there was a deviation between the actual force and the reference force *P*_ref_, as shown in Figure 8(a). This result is also confirmed by Figure 8(b). To eliminate the deviation between the actual force and the reference force, the angle of the chest holder was adjusted to be smaller than the original angle. The adjusted posture of the chest holder was more horizontal than the original posture. The change of the angle of the chest holder also directly affected the forces acting on the experimenter.  Figure 8(c) demonstrated that the adjusted motion increased the force acting on the chest of the experimenter, compared with the original motion. As the adjusted chest holder supported more weight of the experimenter, the force acting on the experimenter's armpits was reduced. Because the robot only supported the chest and armpits of the experimenter in carrying, the friction between the experimenter and the chest holder was ignored since there is no relative motion. The combined force of the forces acting on the chest and armpits equals gravity, and an increase in the force acting on the chest led to a decrease in forces acting on the armpits. Moreover, the experimenter's armpit was more sensitive to feelings than his/her chest. A reasonable reduction in the forces acting on armpits made it easier to ensure the comfort of the experimenter. The experimenter's subjective feeling also demonstrated that the adjusted motion is more comfortable than the original with the reduction of the force acting on the armpits.

The comfort of the experimenters is the most direct way to verify the effectiveness of the motion adjustment method. A sensory test was used to record the feelings of the experimenters. The sensory test had three stages: discomfort, moderation, and comfort [31–35]. The evaluation items were the feeling of the chest, the feeling of the armpits, and the overall evaluation. Table 3 shows the subjective evaluation results of the 10 experimenters. These were the experimenters' evaluations of the overall feeling of being carried.

Comparing the results of the original motion and the adjusted motion, the authors found that the experimenters had different subjective feelings. Most of the experimenters had a negative attitude towards carrying with the original motion because of discomfort in their armpits. The original motion was designed by interpolating several designated postures. The angle of the chest holder cannot be adjusted in lifting with the original motion. It is difficult to make a robot adapt to the body of experimenters because it has a high impact on the body of experimenters during carrying, especially when the experimenters suddenly change their posture. However, the carrying motion with adjusted motion has the opposite result. During carrying, the chest holder can be well adapted to the experimenter's body by adjusting its motion according to the pressure of the experimenter's chest. In addition, the chest holder supports most of the weight of the experimenter and reduces the pressure of their armpits in carrying. Most of the experimenters considered lifting with the adjusted motion to be comfortable. The large contact area between the subject's chest and the chest holder made the experimenter's chest insensitive to uncomfortable feelings. Therefore, although the adjusted motion increases the experimenter's chest pressure, the subject's comfort is improved due to the reduced pressure on the armpits.

## 5. Conclusion

To achieve a comfortable lift for care-receivers when a care-receiver changes his/her posture suddenly, the authors proposed a tactile-based motion adjustment method using active impedance control for a patient transfer robot. This method can adjust the chest holder angle of the robot by measuring the pressure from the chest acting on the chest holder using a tactile sensor mounted on the chest holder. A human-robot dynamic model was built. A dynamic simulation and a test of six subjects were conducted to verify that the angle of the chest holder is a significant factor affecting the comfort of care-receivers. Moreover, another experiment was also conducted to verify the effectiveness of the motion adjustment method.

A dynamic simulation and a test of six subjects confirmed that the angle adjustment of the chest holder is the most effective way to ensure the comfort of a care-receiver compared to horizontal and vertical displacement. In addition, experiments were conducted with 10 subjects (3 females and 7 males), and it was confirmed that the proposed motion adjustment method is effective in ensuring the comfort of the care-receiver. This method helps to reduce the impact and pressure concentration acting on the body of a care-receiver. According to the subjective feelings of experimenters, the method made the experimenters to feel more comfortable than without using the method.

Future studies will include the optimization of control parameters. This will contribute to improving the comfort of service robots.

## Figures and Tables

**Table 1:** Basic specifications of robot.

Item		Value
(A) Dimensions (mm)	(a) Width	755
	(b) Depth	1160
	(c) Maximum height	1964

(B) Weight (kg)		162

(C) Degree of freedom	(a) Chest holder	3
	(b) Cart	4
	(c) Saddle	1
	(d) Paddle	1
	(e) Antioverturning device	1

(D) Maximum load (kg)		90

**Table 2:** The simulation models and subjects parameters.

Model	Height (mm)	Weight (kg)	Subject	Gender	Height (mm)	Weight (kg)
M 1	1700	75	S1	Male	1700	76.5
			S2	Male	1700	76

M 2	1750	75	S3	Male	1750	75.5
			S4	Male	1760	76

M 3	1800	75	S5	Male	1790	81
			S6	Male	1810	73

**Table 3:** Experimenter's feelings during experiment.

Experimenter	Gender	Height (mm)	Weight (kg)	Subjective feelings	
				Original motion	Adjustment motion
E 1	Female	1670	54	×	○
E 2	Female	1680	60	×	○
E 3	Female	1690	65	×	○
E 4	Male	1700	62	×	○
E 5	Male	1700	76	×	○
E 6	Male	1750	70	×	○
E 7	Male	1750	61	×	○
E 8	Male	1770	81	×	○
E 9	Male	1795	81	×	○
E 10	Male	1810	73	×	○

**Table 4:** The impedance parameter in the experiment.

Symbol	Value
*M*_*d*_	0.001 kPa
*B*_*d*_	0.5 kPa/s
*K*_*d*_	4 kPa/s^2^

## Data Availability

The data used to support the findings of this study are included within the article.

## References

[1] Sefcik J. S., Johnson M. J., Yim M. (2018). Stakeholders’ perceptions sought to inform the development of a low-cost mobile robot for older adults: a qualitative descriptive study. *Clinical Nursing Research*.

[2] Zsiga K., Tóth A., Pilissy T. (2017). Evaluation of a companion robot based on field tests with single older adults in their homes. *Assistive Technology*.

[3] Phannil N., Jettanasen C. (2021). Design of a personal mobility device for elderly users. *Journal of Healthcare Engineering*.

[4] Chen N., Song J., Li B. (2019). Providing aging adults social robots’ companionship in home-based elder care. *Journal of Healthcare Engineering*.

[5] Zhou B., Wu K., Lv P. (2018). A new remote health-care system based on moving robot intended for the elderly at home. *Journal of Healthcare Engineering*.

[6] Harling M., Schablon A., Nienhaus A. (2013). Validation of the German version of the nurse-work instability scale: baseline survey findings of a prospective study of a cohort of geriatric care workers. *Journal of Occupational Medicine and Toxicology*.

[7] Greenhalgh B. S. M., Landis B. S. J. M., Brown B. S. J. (2019). Assessment of usability and task load demand using a robotic assisted transfer device compared to a Hoyer advance for dependent wheelchair transfers. *American Journal of Physical Medicine & Rehabilitation*.

[8] Kume Y., Kawakami H. (2008). Development of power-motion assist technology for transfer assist robot. *Matsushita Technical Journal*.

[9] Futoshi Y., Kenichiro H., Kengo N. (2015). Transferring machine for caregiving.

[10] Kume Y., Tsukada S., Kawakami H. (2015). Design and evaluation of rise assisting bed “resyone” based on ISO 13482. *Journal of the Robotics Society of Japan*.

[11] Insider R. (2015). *SASUKE and LOVE to Serve Medical Activities*.

[12] Schoenfisch A. L., Lipscomb H. J., Pompeii L. A., Myers D. J., Dement J. M. (2013). Musculoskeletal injuries among hospital patient care staff before and after implementation of patient lift and transfer equipment. *Scandinavian Journal of Work, Environment & Health*.

[13] Ding J., Lim Y. J., Solano M. Giving patients a lift—the robotic nursing assistant (RoNA).

[14] Mukai T., Hirano S., Nakashima H. Development of a nursing-care assistant robot RIBA that can lift a human in its arms.

[15] Toyota Motor Co., Ltd (1933). *A Patient Transfer Assist Robot*.

[16] Fuji Machinery Co., Ltd (2013). *A Nursing Care Robot*.

[17] Liu Y., Chen G., Liu J. Biomimetic design of a chest carrying nursing-care robot for transfer task.

[18] Faucher M., Brulotte D. A. (2016). Patient/invalid lift with support line bearing power and data communications.

[19] Kawakami H., Kume Y., Nakamura T. (2009). Development of transfer assist robot. *Journal of the Robotics Society of Japan*.

[20] https://www.tekscan.com/TEKSCAN,

[21] Sharifi M., Salarieh H., Behzadipour S. (2018). Patient-robot-therapist collaboration using resistive impedance controlled tele-robotic systems subjected to time delays. *ASME Journal of Mechanical Design*.

[22] Kani M. H. H. M. A., Bonabi H. A. Y., Bidgoly H. J. (2016). Design and implementation of a distributed variable impedance actuator using parallel linear springs. *ASME Journal of Mechanical Design*.

[23] Sato A., Funabora Y., Doki S. Compliant control system based on contact force distribution for wearable robot with tactile sensors.

[24] Han T., Funabora Y., Doki S., Doki K. Improvement of safety by pressure distribution based compliant control for human cooperative robot.

[25] Chu Z., Zhou M., Hu J., Lu S. (2014). Gripping mode analysis of an active-passive composited driving self-adaptive gripper mechanism. *Acta Aeronautica et Astronautica Sinica*.

[26] Ajoudani A., Godfrey S. B., Bianchi M. (2014). Exploring teleimpedance and tactile feedback for intuitive control of the pisa/IIT SoftHand. *IEEE Transactions on Haptics*.

[27] Kuehn J., Haddadin S. (2016). An artificial robot nervous system to teach robots how to feel pain and reflexively react to potentially damaging contacts. *IEEE Robotics and Automation Letters*.

[28] Guadarrama-Olvera J. R., Dean-Leon E., Bergner F., Cheng G. (2019). Pressure-driven body compliance using robot skin. *IEEE Robotics and Automation Letters*.

[29] Chu Z.-Y., Yan S.-B., Hu J., Lu S. (2018). Impedance identification using tactile sensing and its adaptation for an underactuated gripper manipulation. *International Journal of Control, Automation and Systems*.

[30] Liu Y., Guo S., Chen G., Liu J., Gan Z. (2020). Bionic motion planning and the analysis for human comfort of a piggyback nursing-care robot for transfer tasks. *Chinese Journal of Mechanical Engineering*.

[31] Wang T., Jeong H., Watanabe M., Iwatani Y., Ohno Y. (2018). Fault classification with discriminant analysis during sit-to-stand movement assisted by a nursing care robot. *Mechanical Systems and Signal Processing*.

[32] Goncalves R., Hamilton T., Daher A. MIT-skywalker: evaluating comfort of bicycle/saddle seat.

[33] Li X., Ding L., Ma X., Li B., Liu H. Development of a human-seat cushion finite element model for sitting comfort analysis.

[34] Singh R., Leon D., Morrow M. (2016). Effect of chair types on work-related musculoskeletal discomfort during vaginal surgery. *American Journal of Obstetrics and Gynecology*.

[35] Mastrigt S. H., Groenesteijn L., Vink P. (2016). Predicting passenger seat comfort and discomfort on the basis of human, context and seat characteristics: a literature review. *Ergonomics*.

